# Study of the Antioxidant Capacity and Oxidation Products of Resveratrol in Soybean Oil

**DOI:** 10.3390/foods13010029

**Published:** 2023-12-20

**Authors:** Yunping Yao, Huiping Yuan, Chen Chen, Jia Liang, Changmo Li

**Affiliations:** State Key Laboratory of Food Nutrition and Safety, Key Laboratory of Food Nutrition and Safety, Ministry of Education, College of Food Science and Engineering, Tianjin University of Science and Technology, Tianjin 300457, China; yaoyunping@tust.edu.cn (Y.Y.); yuanhp14@163.com (H.Y.); 18404982495@163.com (C.C.); liangjia202312@163.com (J.L.)

**Keywords:** resveratrol, free radical scavenging, oxidative cracking, oxidation products, stability

## Abstract

Resveratrol (3,5,4′-trihydroxystilbene), a naturally occurring polyphenol that is widely utilized in functional food due to its antioxidant, anti-inflammatory, anti-cancer and anti-aging properties. In the present study, the antioxidant capacity and oxidation products of resveratrol in soybean oil were investigated. The antioxidant activity of resveratrol was evaluated by employing various in vitro antioxidant assays such as DPPH scavenging activities, ferric reducing abilities (FRAP) and oxygen radical absorbance capacity (ORAC). Furthermore, monitoring the peroxide value and the acid value of soybean oil with the addition of 200–1000 μg/g of resveratrol at 60 and 180 °C. It was found that when the concentration of resveratrol in soybean oil was 600 µg/g, the antioxidant capacity was most effective. Resveratrol and its thermal degradation products were identified using liquid chromatography–mass spectrometry (LC–MS) and gas chromatography–mass spectrometry (GC–MS). There were seven nonvolatile oxidation products with mass-to-charge ratios of 138.03, 171.04, 185.10, 157.03, 436.13, 244.07 and 306.09 kg/C and two volatile oxidation products with mass-to-charge ratios of 100.05 and 158.13 kg/C were identified. The research findings may provide essential information for the development of resveratrol as functional oils in future.

## 1. Introduction

Resveratrol is a non-flavonoid polyphenolic compound with a stilbene structure. It is widely found in natural plants or fruits such as grapes, pine, cinnamon and peanuts [1]. Resveratrol is found in two isoforms: trans-resveratrol and cis-resveratrol. The trans isomer has a higher biological activity due to the presence of the 4′-hydroxystyryl group [2]. Resveratrol is well-known for its multiple pharmacological activities, such as being anti-inflammatory, antioxidant, antimicrobial, anticancer, neuroprotective and cardioprotective effects [3,4,5]. Definite pharmacological activities have led to supplement and food products containing resveratrol and its emergence as a promising new health ingredient.

In food applications, consumers are increasingly interested in natural and safe antioxidants. Therefore, there are increasing studies of resveratrol as a natural antioxidant for maintaining nutritional quality and replacing of synthetic antioxidants (BHA, BHT and TBHQ). But the structure of resveratrol is prone to degradation and isomerization under specific conditions, such as alkaline conditions [6]. Furthermore, due to the photosensitivity of resveratrol, its trans isomer can isomerize to cis-resveratrol under ultraviolet or visible light [7]. Resveratrol is sensitive to light, pH and increased temperature due to its unstable hydroxyls and the double C–C bond [6]. Currently, reports on the stability of resveratrol compounds are predominantly focused on photostability [8,9]. Hernández’ experiments showed that resveratrol is oxidized in the presence of UV irradiation to produce a photo-oxidation product with a mass-to-charge ratio of 212, but the pathway of oxidative cleavage of this product has not been resolved [10].

As an antioxidant of nature, resveratrol had a positive impact on inhibiting lipid peroxidation. Due to the inherent presence of resveratrol in peanuts, current research on the antioxidant properties of resveratrol in oils predominantly focuses on peanut oil [11,12,13]. However, there is little information about the degradation and thermal oxidation products of resveratrol in lipids. Simultaneously, edible oils undergo a complex reaction during processing, which is susceptible to factors such as temperature, oxygen, impurities, etc. [14], which can result in a reduction in the content and biological activity of resveratrol. The limited studies on resveratrol in other commonly used edible oils, also affecting its potential as an excellent antioxidant. Therefore, for a more effective application of resveratrol in the field of edible oil antioxidants, it is necessary to assess the stability of resveratrol during simulated accelerated oxidation or high-temperature frying and its impact on the quality of soybean oil.

The objective of this study is to assess the antioxidant capacity of resveratrol and its impact on the oxidative stability of soybean oil. Furthermore, the thermal oxidative degradation products of resveratrol at 60 and 180 °C were identified by HPLC-MS and GC-MS, and a comprehensive investigation of the dynamic changes of resveratrol and its oxidation products was conducted. These findings will provide theoretical insights and guidance for the development and use of functional edible oils.

## 2. Materials and Methods

### 2.1. Materials

Soybean oil was purchased from the local market. Resveratrol (purity ≥ 99%) was obtained from Tianjin Solomon Biotechnology Co., Ltd. (Tianjin, China). In addition, 1,1-diphenyl-2-picryl-hydrazyl (DPPH), 6-hydroxy-2,5,7,8-tetramethyltryptamine-2- carboxylic acid (Trolox) and 2,4, 6-tri (2-pyridyl) triazine (TPTZ) were purchased from Shanghai Yuanyiye Biological Co., Ltd. (Shanghai, China). HPLC-grade solvent methanol was purchased from Thermo Fisher (Waltham, MA, USA).

### 2.2. Sample Preparation

Resveratrol was added to soybean oil to obtain oil samples, with concentrations of 200, 400, 600, 800 and 1000 µg/g of resveratrol. The samples were then subjected to ultrasound treatment for 10 min to ensure complete dispersion of resveratrol in the oil. The oil samples were placed in a 9 mm Petri dish and heated for 0–12 d at 60 °C and 0.5–8 h at 180 °C. The oxidized samples were removed periodically from the oven and stored at −20 °C until analysis was performed.

### 2.3. Determination of Antioxidant Capacity of Resveratrol

#### 2.3.1. DPPH Free Radical Scavenging Activity

The DPPH method was determined according to the previous method with some modifications [15]. First, 500 µL of diluted samples or standard Trolox solution (20, 40, 100, 200, 300 µmol/L) was mixed with 300 µL of the DPPH solution (60 µmol/L) and incubated in the dark for 30 min. The absorbance was measured at 517 nm with methanol as a blank sample. The scavenging capacity of resveratrol against DPPH-free radicals was expressed in terms of Trolox equivalents (µmol/L TE).

Elimination rate% = [1 − (A_1_ − A_2_)/A_C_] × 100%

A_C_: Absorbance of the DPPH and solvent mixture; A_1_: Absorbance of the DPPH after reaction with the sample; A_2_: Absorbance of the sample and solvent mixture.

#### 2.3.2. Ferric-Reducing Antioxidant Power (FRAP)

The FRAP method was conducted according to the previous method with some modifications [16]. HCl (40 mmol/L) and 2,4,6-tripyridyltriazine (TPTZ) reagent were mixed to prepare a 10 mmol/L TPTZ stock solution. A mix of 25 mL of acetic acid-sodium acetate buffer (PH = 3.6), 2.5 mL of TPTZ solution and 2.5 mL of FeCl_3_ solution (20 mmol/L) was prepared at 37 °C for use as the working solution. Take the blank and sample filtrate to measure its absorbance value at 593 nm. The ferric-reducing antioxidant power of resveratrol was expressed in terms of Trolox equivalents (µmol/L TE).

#### 2.3.3. Oxygen Radical Absorbance Capacity (ORAC)

The ORAC was conducted according to the previous method with some modifications [17]. First, 25 µL of resveratrol was added to a 96-well black microplate and stored at 37 °C for 20 min. Then, 25 µL of AAPH solution (153 mmol/L) was added with a multichannel pipette to rapidly initiate the reaction. The excitation wavelength was set at 485 nm, the emission wavelength was set at 538 nm and the fluorescence intensity was continuously measured. During the measurements, the system temperature was maintained at 37 °C and the fluorescence intensity was measured every 2 min. Before each measurement, the fluorescence intensity was shaken at medium speed for 10 s. The measurement was stopped after the fluorescence intensity decayed to baseline. The ORAC value of resveratrol was calculated by integrating the area under the fluorescence decay curve.

$$\mathrm{ORAC}=\frac{{AUC}_{S}-{AUC}_{C}}{{AUC}_{Trolox}-{AUC}_{C}}\times \frac{C_{Trolox}}{C_{Sample}}$$

*AUC_S_*: Area under the fluorescence decay curve with the addition of the sample; *AUC_C_*: Area under the fluorescence decay curve without the addition of an antioxidant; *AUC_Trolox_*: Area under the fluorescence decay curve with the addition of Trolox; *C_Trolox_*: Trolox concentration; *C_Sample_*: Sample concentration.

#### 2.3.4. Analysis of Peroxide Value and Acid Value

The peroxide value was determined using the official methods of AOCS Cd 8b-90. Generally, 2 g of oxidized sample was weighted in a volumetric flask (250 mL), then a 30 mL mixture of chloroform and glacial acetic acid (2:3, *v*/*v*) was added, the flask was shaken and then add 1.0 mL of saturated potassium iodide solution. Subsequently, it was plugged immediately, gently shaken for 30 s and placed it in a dark place for 3 min, after which 100 mL of distilled water was added. After shaking, the mixture was titrated with a standard sodium thiosulfate solution. Then, 1 mL of starch indicator was added when the solution color became light yellow, followed by continued titration until the blue color disappeared, which was the end point of the titration. The blank assay was measured under the same conditions. POV was expressed as the value of millimoles of reactive oxygen species in 1 kg sample (mmol/kg) and calculated according to the following equation:

POV = (V − V_0_) × c/(2 × m) × 1000

where V and V_0_ are the volume of the sodium thiosulfate standard solution consumed by the sample titration and the blank assay (mL), c is the concentration of the sodium thiosulfate standard solution, m is the weight of the sample (g) and 1000 is the conversion factor.

The acid value was determined using the official methods of AOCS Cd 3d-63. First, an appropriate mass of the sample was weighed and placed it in a conical flask and 50 mL of the ethyl ether-isopropyl alcohol mixture (1:1, *v*/*v*) was added, as well as 3 to 4 drops of the phenolphthalein indicator of. The mixture was shaken thoroughly to dissolve the sample and then titrated with a standard potassium hydroxide titration solution. The sample solution first appeared slightly pink and there was no apparent fading within 15 s. The volume (in milliliters) of the standard titration solution consumed at this endpoint was recorded; this value is denoted as V. The acid value is calculated according to the following formula:

AV = (V − V_0_) × c × 56.1/m

where V and V_0_ represent the volumes of standard titration solution consumed in the sample titration and the blank test (mL), respectively; c is the concentration of the standard titration solution (mol/L), m is the weight of the sample (g) and 56.1 is the molar mass of potassium hydroxide.

### 2.4. Thermogravimetric Analysis

Thermogravimetric analysis (TGA, Netzsch, GER) was used to investigate the thermal stability and composition of materials by measuring the relationship between the mass of the sample and the temperature under controlled conditions [18]. In this study, the thermal stability of trans-resveratrol was determined using a TGA instrument. Approximately 5 mg of untreated trans-resveratrol standard was placed in a crucible and the temperature was programmed as follows: a 2-min ramp from room temperature to 35 °C, followed by a 900 s equilibration to ensure uniformity between the furnace and sample temperatures. The temperature was then increased at a rate of 5 °C/min in a nitrogen atmosphere until it reached 600 °C [19].

### 2.5. Determination of Resveratrol in Oil

The liquid–liquid extraction method was used for the extraction of resveratrol from oil, using a 75% ethanol solution with ultrasonic extraction for 50 min. The HPLC determination conditions of resveratrol were performed according to the method with some modifications [20]. High-performance liquid chromatography (HPLC) was performed using a Shimadzu LC-20AT liquid chromatograph equipped with an SPD-M20A diode array detector (Shimadzu, Kyoto, Japan), with a detection range of 200–800 nm. Separations were carried out with an Agela Innoval C18 column (4.6 mm × 250 mm, 5 µm, Agilent Technologies). The mobile phase was a gradient of ultrapure water with methanol (0 min, 65:35; 5 min, 40:60; 11 min, 10:90; 15 min, 0:100; 16 min, 65:35; 25 min, 65:35). Analysis was carried out at a flow rate of 1 mL/min at the detection wavelength of 310 nm with a column heater temperature of 35 °C and the injection volume of the sample was 10 µL.

### 2.6. Analysis of Resveratrol Oxidation Products

#### 2.6.1. Identification of Non-Volatile Thermal Oxidation Products by LC-MS

The conditions for high-performance liquid chromatography were consistent with the aforementioned Section 2.5. The analysis protocol was carried out on a Shimadzu LC-MS instrument (ESI-IT-TOF) with scanning performed in both positive and negative ion modes. The mass spectrometry scanning range was set between 90 and 1000 *m*/*z*. Other MS parameters, modified according to the method outlined by Wang et al. [21], included ESI as the ion source, with detector and interface voltages set at 1.57 and 4.5 kV, respectively. The capillary discharge line (CDL) and heating block temperatures were maintained at 200 °C. Nitrogen gas, at a flow rate of 1.5 L/min, was utilized as the nebulizing gas, and the mobile phase consisted of methanol and water with a flow rate of 0.2 mL/min.

#### 2.6.2. Identification of Volatile Thermal Oxidation Products by GC-MS

Volatile compounds were analyzed by headspace solid phase microextraction (SPME), and the method was slightly modified based on previous reports [22]. All extractions use divinylbenzene/carboxyl/polydimethylsiloxane (DVB/CAR/PDMS) fiber (50/30 µm, Anpel Laboratory Technologies, Shanghai, China). The oxidized oil (4 g) was accurately weighed in a 25 mL headspace bottle and equilibrated on a magnetic stirring heater at 80 °C for 20 min. It was then extracted for 30 min and desorbed for 10 min.

All analyses were performed on an Agilent 7890B gas chromatography system (Agilent Technologies, Santa Clara, CA, USA) equipped with an Agilent 5977A mass spectrometer. Compound separation was performed on an HP-5 fused silica capillary column (30 m × 0.25 mm, 0.25 µm, Agilent Technologies) [23]. The heating procedure is as follows: the initial temperature was kept at 40 °C for 3 min, then at a rate of 6 °C/min to 160 °C and kept at 160 °C for 1 min. Finally, the temperature was increased to 250 °C at a rate of 8 °C/min for 6 min. In addition, the split ratio was 5:1 and the inlet temperature was 270 °C. Helium is used as the carrier gas with a flow rate of 1.5 mL/min. The temperature of the mass spectrometer (MS) source and the interface is 230 and 250 °C, respectively. The mass spectrum is scanned from 30 to 800 *m*/*z*. An electron impact (EI) of 70 eV was used for ionization [24].

### 2.7. Statistical Analysis

All tests were carried out in triplicate. Results were expressed as the Mean ± SD. Origin 9.0 and Chem Draw 19.0 software were used to process and plot the data.

## 3. Results

### 3.1. In Vitro Antioxidant Capacity of Resveratrol

**Figure 1:**
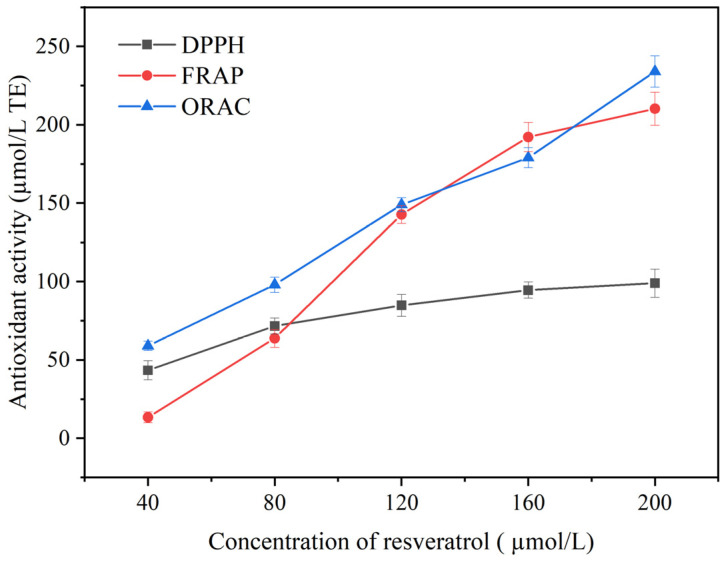
In vitro antioxidant capacity of resveratrol.

This study used several different analytical techniques, such as DPPH, FRAP and ORAC, to measure the antioxidant activity of resveratrol (Figure 1). These methods included two types of tests [25]: the first in which a single electron transfer reaction was monitored by the color change that occurred as the oxidant was reduced (DPPH and FRAP assays) and the second based on a hydrogen atom transfer reaction, in which the antioxidant and the substrate (probe) compete for free radicals (ORAC method). The detection results for the three antioxidant capacities in vitro all exhibited a significant dose–response relationship between the concentration of resveratrol and its antioxidant activity. Furthermore, resveratrol demonstrated the highest antioxidant activity in the ORAC assay.

For the scavenging capacity of DPPH free radicals (Figure 1), this may indicate that resveratrol can reduce the activity of free radicals by neutralizing the DPPH radicals through electron and/or hydrogen donors, and prevent further chain reaction of free radicals, thus achieving the effect of scavenging DPPH free radicals [26]. Wei et al. investigated the antioxidant activity of parent resveratrol molecules and their derivatives using electron paramagnetic resonance (EPR). The results indicated that resveratrol exhibited a significant ability to eliminate DPPH radicals [27]. In the FRAP assay, the antioxidant activity of resveratrol was characterized by the ability to directly reduce Fe (III) to Fe (II), and the antioxidant capacity increased from 13.42 to 210.26 µmol/L TE (15.67 folds) with increasing concentration of resveratrol (Figure 1). The special position of the hydroxyl group is the primary factor that determines its chelating ability with metal ions. Stivala et al. investigated the antioxidant properties of resveratrol and its analogues from the perspectives of stereoisomerism, position of the hydroxyl group and presence of double bonds [28]. They suggested that 4′-OH is not the only factor that influences its antioxidant activity, and there is also a synergistic effect between 3′-OH and 5′-OH. ORAC takes into account the action of antioxidants on the inhibition time and the percentage of inhibition of peroxyl radicals, which are the most abundant radicals in biological systems [29]. And, resveratrol exhibits the highest antioxidant activity in the ORAC assay (Figure 1). Cheng et al., demonstrated that resveratrol exerted a protective effect on the heart and blood vessels by directly scavenging oxygen radicals, thus preventing oxidative stress-induced endothelial apoptosis [30]. A study by He et al., also demonstrated that the inhibitory effect of resveratrol on osteoclasts was achieved by suppressing peroxide radicals, and it showed a dose–response relationship [31]. Furthermore, studies have indicated that radical scavenging activity may depend on the number and position of hydroxy groups [32].

### 3.2. Antioxidant Capacity of Resveratrol in Oil

**Figure 2:**
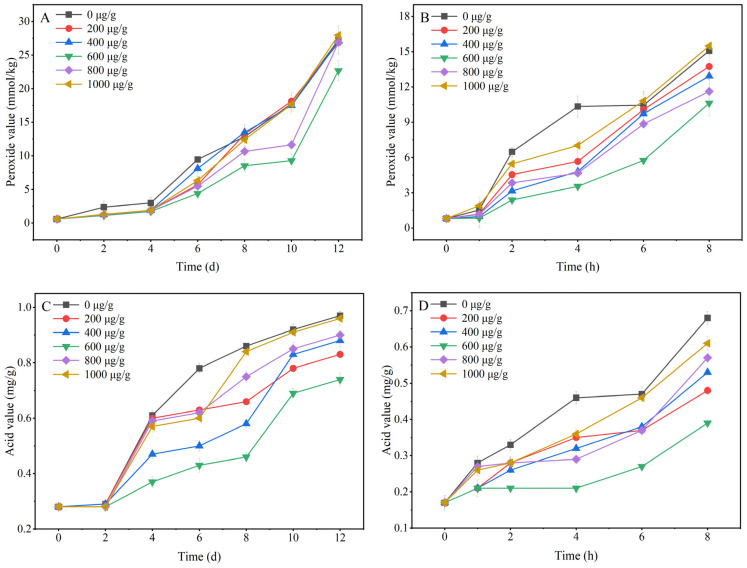
(**A**) Changes of peroxide value of resveratrol during heating at 60 °C. (**B**) Changes of peroxide value of resveratrol during heating at 180 °C. (**C**) Changes of acid value of resveratrol during heating at 60 °C. (**D**) Changes of acid value of resveratrol during heating at 180 °C.

Figure 2 showed the changes in the peroxide value and the acid value during the oxidation of different concentrations of resveratrol added to soybean oil at 60 and 180 °C. Under both heating conditions, the peroxide values of the control soybean oil and resveratrol-added soybean oil increased with increasing heating time (Figure 2A,B). This indicates that during the oxidation process in this experiment, the formation of hydroperoxides in soybean oil was significantly higher than the removal capacity of antioxidants and the degradation rate of peroxides, resulting in a gradual high oxidation of soybean oil during the heating process. However, there were differences in the effects of different concentrations of resveratrol on the degree of oil oxidation.

When the heating condition was 60 °C, the peroxide values of the samples with each spiked concentration were lower than those of the control group within 6 d of heating (Figure 2A). When the heating lasted for 8 d, the peroxide values of the samples containing spiked concentrations of 200, 400 and 1000 µg/g exhibited a sharp increase, approaching those of the control group. In particular, the samples with a concentration of 600 µg/g consistently showed the lowest peroxide levels throughout the 12 days heating process. At a heating temperature of 180 °C, the peroxide values of the samples in the initial stage of oxidation (0–1 h) were not significantly different from those of the control group, and after 2 h of oxidation, the peroxide values of the control group increased sharply (Figure 2B). At 6 h of heating, the peroxide value of the sample with 1000 µg/g was slightly higher than that of the control group, indicating that the high concentration of resveratrol had started to lose its antioxidant effect at this time. The peroxidation value of the sample with 600 µg/g was always the lowest throughout the 8 h heating period, which was the same as that of the heating at 60 °C.

The reason for this phenomenon could be that, at low concentrations, resveratrol provides electron and/or hydrogen donors through the three hydroxyl groups carried by its own structure to scavenge free radicals [33]. However, when the concentration of resveratrol in the system was greater than 800 µg/g, the metal ions that could be present in the system were able to reduce excess resveratrol to generate reactive oxygen species (ROS) and phenol oxygen radicals and therefore the antioxidant properties of resveratrol were reduced. When the samples were heated for too long under high temperature conditions, there were changes in the position and number of resveratrol hydroxyl groups [34], double bonds were reduced and the monomers were oxidized and polymerized to epoxy compounds [35], releasing alkoxy groups and the antioxidant capacity was significantly weakened.

Furthermore, the trends in acid value and peroxide value of different concentrations of resveratrol remained consistent under both heating conditions (Figure 2C,D). However, the acid values of the samples with a concentration of 1000 µg/g were lower than those of the control group and the peroxide values were higher than those of the control group during 0–12 d of heating at 60 °C and 0–8 h of heating at 180 °C. This indicates that although the double bonds of unsaturated fatty acids were opened and a large number of primary peroxides were gradually formed after prolonged heating of the samples with high resveratrol addition. These primary peroxides were not yet fully oxidized further to aldehydes, ketones and acid secondary oxidation products, resulting in an increase in free fatty acids decomposed at this time that was still lower than that of the control group. Furthermore, in the range of 200–1000 µg/g, the acid value and peroxide value of the sample with 600 µg/g were always the lowest value, implying that the addition of 600 µg/g of resveratrol to soybean oil can effectively inhibit the production of peroxide and unsaturated fatty acids.

### 3.3. Thermal Stability of Resveratrol

#### 3.3.1. Thermogravimetric Analysis

**Figure 3:**
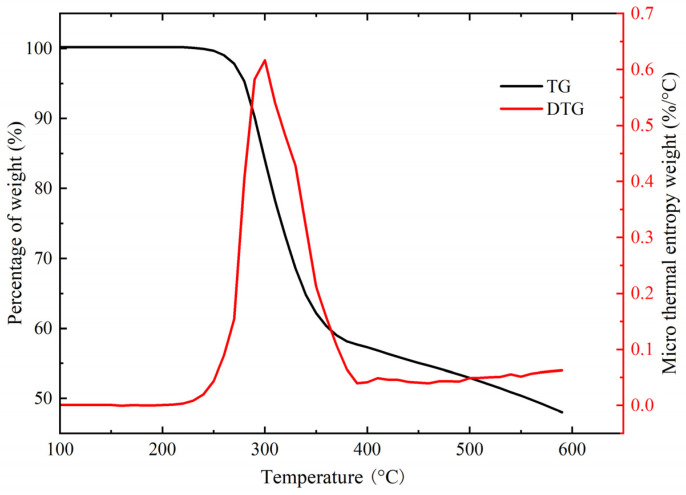
Thermogravimetric degradation of resveratrol.

The thermal degradation curves of the trans-resveratrol standards are shown in Figure 3. Resveratrol mass loss can be divided into three parts: the first is the water loss stage, the second is the thermal decomposition and polymerization stage, and the third is the carbonization stage [36]. Before reaching 240 °C, the TG (thermogravimetric curve) and DTG (differential thermogravimetric curve) of the resveratrol remained level and its mass fraction was constant at 100%, indicating that no decomposition of the resveratrol crystals occurred before that and no water was adsorbed in the crystals. After reaching 240 °C, the resveratrol began to decompose, the mass fraction began to decrease, the DTG curve leveled off at 458 °C and its value tended toward zero, and the decomposition process ended, at which time the mass fraction of the remaining resveratrol was 53.31%. Combined with the experimental weight loss rate of 46.69% measured by the TG curve, it was inferred that the weight loss was caused by the breakage of the C2–C7 chemical bond in the resveratrol molecule.

#### 3.3.2. Degradation of Resveratrol during Heating in Oils

**Figure 4:**
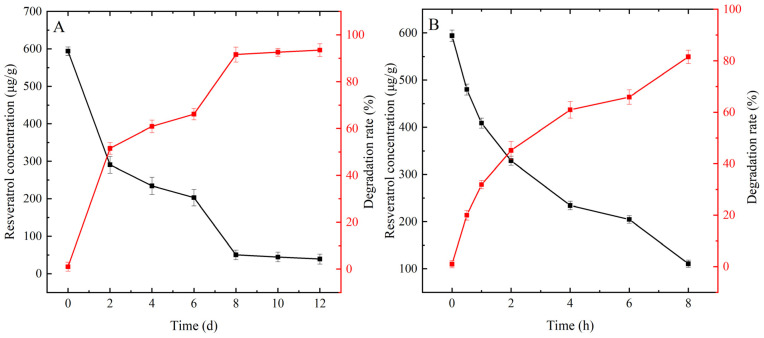
(**A**) Degradation of resveratrol at 60 °C. (**B**) Degradation of resveratrol at 180 °C.

The sample with a concentration of 600 µg/g of resveratrol exhibited the best antioxidant ability in terms of peroxide value and acid value in the experiments. Therefore, to better understand the changes in resveratrol during the oxidation process, the thermal degradation rates of 600 µg/g of resveratrol were investigated at 60 and 180 °C. The resveratrol content gradually decreased with increasing oxidation time. At 60 °C, the resveratrol dose reached 50% after 3 d of heating. The consumption of resveratrol was 93.5% after heating to 12 d (Figure 4A). After 2 h of heating at 180 °C, the degradation rate of resveratrol reached 45.17%. Furthermore, the degradation rate increased to 81.54% after 8 h of heating (Figure 4B). Upon calculation, the degradation rates of resveratrol at both temperatures conform to a first-order reaction kinetic model, with correlation coefficients for the rate constants (k) being 2.341 × 10^−1^ d^−1^ (R^2^ = 0.8912), 1.769 × 10^−1^ h^−1^ (R^2^ = 0.9677), respectively.

### 3.4. Identification of Oxidation Products

**Figure 5:**
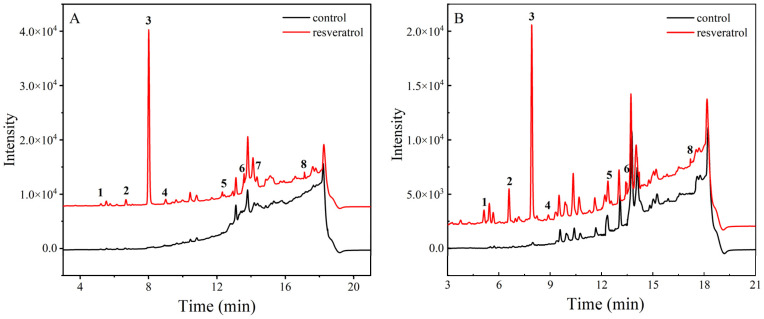
(**A**) Chromatogram of non-volatile oxidation products of resveratrol at 60 °C. (**B**) Chromatogram of non-volatile oxidation products of resveratrol at 180 °C.

As can be seen in Figure 5, compared to the control sample under the same heating conditions, the sample spiked with resveratrol showed several additional peaks (Figure 5A peaks **1**, **2**, **4**, **5**, **6**, **7** and **8**) in addition to the target resveratrol (Figure 5A peak **3**) after heating at 60 °C. The sample heated at 180 °C generated additional peaks **1**, **2**, **4**, **5**, **6** and **8** compared to the control (Figure 5B), indicating that new oxidation products were generated by the degradation of the resveratrol during oxidation.

Resveratrol contains phenolic hydroxyl groups that readily lose a proton to obtain [M − H]^−^ excimer ions. In the negative ion scan mode of LC–MS, the full first-level mass spectrum of resveratrol (peak **3**) was based on the excimer ion peak *m*/*z* 227 [M − H]^−^ without polymerization or addition of ions. The product ions of (**1**) *m*/*z* 138.03, (**2**) *m*/*z* 171.04, (**4**) *m*/*z* 185.10, (**5**) *m*/*z* 157.07, (**6**) *m*/*z* 436.13, (**7**) *m*/*z* 244.07 and (**8**) *m*/*z* 306.09 were obtained for the unidentified oxidation products after a full scan of positive and negative ions, respectively (Table 1). The mass spectrum of each nonvolatile oxidation product of resveratrol is shown in Figure S1.

In this experiment, the structure of product peak **1** (3,5-dihydroxybenzaldehyde, *m*/*z* 138.03) was derived from the oxidative breakage of the double bond connecting two benzene rings in the structure of resveratrol, and 4′-OH was shed to form hydroxyl radicals and attached to the breakage of the double bond of styrene, leading to the generation of 3,5-dihydroxybenzaldehyde as an oxidation product. And the fragment ion peak of *m*/*z* 90 was found in the resveratrol mass spectrometric fragment ions, and it was speculated that resveratrol formed two partial fragment ions of 3,5-dihydroxybenzaldehyde (*m*/*z* 138.03) and *m*/*z* 90 due to double bond break during the oxidation process [37].

Saturated cyclic compounds are relatively stable in structure and the breaking of a fragment usually requires breaking two chemical bonds, which is a common feature of resveratrol and most other astragalloids when their structures are broken. During heating, a bond break occurs between the two chemical bonds between the 2′ and 3′ positions and the 4′ and 5′ positions of resveratrol, resulting in the loss of one molecule of C_2_H_2_O (42 Da) followed by the formation of an electron-deficient active center at the broken position, which induces the opening of the resorcinols benzene rings to form the product ion at *m*/*z* 185.10 (peak **4**, 4-((1E,4E)-3-methylenehexa-1,4-dien-1-yl)phenol). On this basis, a hydrocarbon group on the carbon atom to which 4′-OH is attached undergoes 1,2-migration with electron pairs, and the unshared electrons on the hydroxyl oxygen atom are transferred between the carbon and oxygen to form a double bond, and the product ion with *m*/*z* 171.04 (peak **2**, 7-hydroxy-1-naphthaldehyde) is formed after cyclization. Meanwhile, the double bond that connects the two benzene ring structures after the opening of the resorcinol ring can no longer form π-π conjugation with phenol, and this double bond under mass spectrometric dissociation conditions is susceptible to cleavage under attack by free radical electrons generated after the loss of C_2_H_2_O fragment, and then the oxidation product of *m*/*z* 157.07 (peak **5**, 2-naphthalenemethanol) is obtained by the cyclization reaction.

Since hydroxyl radicals can initiate three types of oxidation reactions, the extraction of a hydrogen atom, extraction of an electron (charge transfer) or the incorporation of a double bond or an aromatic ring, it is speculated that *m*/*z* 436.13 (peak **6**, dimer1) consists of fragment ions generated by the decomposition of a dimer of two molecules of resveratrol monomer bound in the negative ion mode to form [2M − H]^−^. The polymerization site of this dimer is the oxidative dehydrogenation of the hydroxyl group on a resveratrol resorcinol molecule, and O^−^ attracts electrons on the double bond between the two benzene rings of the other molecule of resveratrol, forming a tetrahydrofuran ring connecting the two molecules of resveratrol after molecular rearrangement [38]. The hydroxyl group of this dimer (*m*/*z* 453) phenol reacts with the hydrocarbon group on the carbon atom of the attached hydroxyl group to generate water, forming the final product ion. However, the tetrahydrofuran ring in the structure of dimer1 is a hydrogenated saturated cyclic compound, which is structurally stable and does not easily polymerize, and has a higher selectivity for hydrogenolysis to open the ring when the reaction occurs. The product ion at *m*/*z* 306.09 (peak **8**, dimer2) is presumed to be generated by the breaking of the C–O ether bond and the breaking of the C–C bond of the tetrahydrofuran ring in the structure of the dimer *m*/*z* 435.

The oxidation product of *m*/*z* 244.07 (peak **7**, 3,4,3′,5′-tetrahydroxy-trans-stilbene) is a molecular ion peak corresponding to the [M + O−1]^−^ of resveratrol by binding a new hydroxyl group to the C atom adjacent to the 4′-OH group of resveratrol. This oxidation product was only produced during the oxidation at 60 °C, and the reason why it was not detected during the oxidation at 180 °C may be due to the secondary decomposition of this oxidation product in the early stage of oxidation during the high temperature oxidation at 180 °C resulting in its non-detection. Moreover, the newly formed neighboring hydroxyl group in its structure can significantly increase the antioxidant activity of resveratrol, so resveratrol is first oxidized to form the more stable 3,4,3′,5′-tetrahydroxy-trans-stilbene during the oxidation process, thus increasing its antioxidant properties.

**Figure 6:**
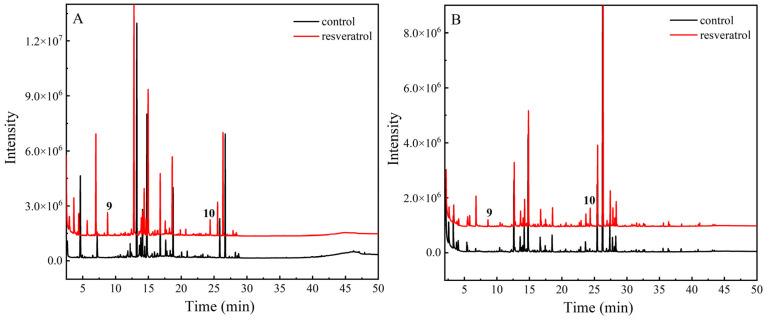
(**A**) Chromatogram of volatile oxidation products of resveratrol at 60 °C. (**B**) Chromatogram of volatile oxidation products of resveratrol at 180 °C.

The soybean oil with 600 µg/g resveratrol and the control group were heated at 60 °C and 180 °C to obtain volatile oxidation products, as shown in Figure 6. Compared to the control group, oil samples with resveratrol addition were oxidized at high temperature to produce two new substances, 2-methyltetrahydrofuran-3-one (peak **9**, *m*/*z* 100.12) and nonanoic acid (peak **10**, *m*/*z* 158.24). Among them, 2-methyltetrahydrofuran-3-one is a benzofuranone formed from dimer1 after removal of three molecules of phenol to retain the dihydroisobenzofuran skeleton and the opening of the ring by breaking two chemical bonds in the benzene ring. Nonanoic acid is formed by opening the oxidative ring of two molecules of phenol of dimer2 after molecular rearrangement. However, traces of nonanoic acid were also found in the control sample after heating. To investigate whether the oxidation product originated from soybean oil itself or was a new oxidation product that could only be formed after the addition of resveratrol, the above experimental procedure was repeated using glycerol palmitate instead of soybean oil, and the data showed that nonanoic acid could not be produced in the heated blank glycerol palmitate, while the sample of glycerol palmitate with resveratrol added contained nonanoic acid. It is deduced that both 2-methyltetrahydrofuran-3-one and nonanoic acid are the oxidation products of resveratrol after heating and oxidation.

### 3.5. Variation in Relative Content of Resveratrol Oxidation Products

**Figure 7:**
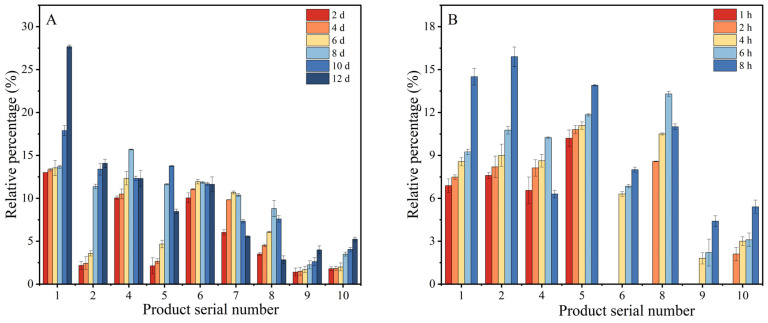
(**A**) Concentration changes of resveratrol oxidation products during heating at 60 °C. (**B**) Concentration changes of resveratrol oxidation products during heating at 180 °C. **1**, 3,5-dihydroxybenzaldehyde. **2**, 7-hydroxy-1-naphthaldehyde. **4**, 4-((1E,4E)-3-methylenehexa-1,4-dien-1-yl)phenol. **5**, 2-naphthalenemethanol. **6**, dimer1. **7**, 3,4,3′,5′-tetrahydroxy-trans-stilbene. **8**, dimer2. **9**, 2-methyltetrahydrofuran-3-one. **10**, nonanoic acid.

The changes in the content of each oxidation product during the heating of resveratrol at 60 °C and 180 °C are shown in Figure 7. 2-methyltetrahydrofuran-3-one were always present simultaneously during heating 0–12 d at 60 °C (Figure 7A). Among them, the relative contents of 3,5-dihydroxybenzaldehyde, 7-hydroxy-1-naphthaldehyde, 2-methyltetrahydrofuran-3-one and nonanoic acid increased with increasing heating time during heating 0–12 d. Additionally, the relative contents of 4-((1E,4E)-3-methylenehexa-1,4-dien-1-yl)phenol, dimer1 and dimer2 all tended to increase and then decrease because they would continue to decompose to produce other oxidation products during the heating process. At day 6 of heating, the degradation rate of resveratrol was 60.93% and the relative content of 3,4,3′,5′-tetrahydroxy-trans-stilbene generated at this time was 10.67%, and then the content of 3,4,3′,5′-tetrahydroxy-trans-stilbene began to decrease, while the degradation rate of resveratrol increased rapidly. It was speculated that the possible reason for this phenomenon was that 3,4,3′,5′-tetrahydroxy-trans-stilbene started to be generated in the early stage of heating and played the main antioxidant role in the system due to the four hydroxyl groups it carried, and with deepening of the oxidation, its consumption gradually exceeded its production and the antioxidant capacity was weakened. Resveratrol continuously supplied hydrogen to the system, accelerating its consumption rate, leading to a more thorough oxidative cleavage and the formation of a significant amount of 7-hydroxy-1-naphthaldehyde, 4-((1E,4E)-3-methylenehexa-1,4-dien-1-yl) phenol, 2-naphthalenemethanol and dimer2.

Under the heating condition of 180 °C, 3,5-dihydroxybenzaldehyde, 7-hydroxy-1-naphthaldehyde, 4-((1E,4E)-3-methylenehexa-1,4-dien-1-yl)phenol and 2-naphthalenemethanol had started to be generated at the early stage of oxidation (1 h), and dimer2 existed in the system at the same time after oxidation up to 4 h (Figure 7B). With the gradual decrease in resveratrol content, the relative content of oxidation products, excluding 4-((1E,4E)-3-methylenehexa-1,4-dien-1-yl)phenol and dimer2, increased steadily with extended heating time. However, the content of 4-((1E,4E)-3-methylenehexa-1,4-dien-1-yl)phenol and dimer2 reached their respective maximum values of 10.24% and 13.3% after 6 h of heating, following which a gradual decreasing trend began to emerge. The possible reason for this phenomenon is that during the heating process, the 4-((1E,4E)-3-methylenehexa-1,4-dien-1-yl)phenol underwent electron rearrangement to gradually oxidize and decompose into 7-hydroxy-1-naphthaldehyde and 2-naphthalenemethanol oxidation products and that the dimer2 decomposed into nonanoic acid from removing the phenol structure. Therefore, the content of 4-((1E,4E)-3-methylenehexa-1,4-dien-1-yl)phenol and dimer2 will appear to rise and then fall during the oxidation process. The relative contents of 7-hydroxy-1-naphthaldehyde, 2-naphthalenemethanol and nonanoic acid, which were generated by the decomposition of 4-((1E,4E)-3-methylenehexa-1,4-dien-1-yl)phenol and dimer2, increased significantly after the contents of 4-((1E,4E)-3-methylenehexa-1,4-dien-1-yl)phenol and dimer2 started to decrease.

**Figure 8:**
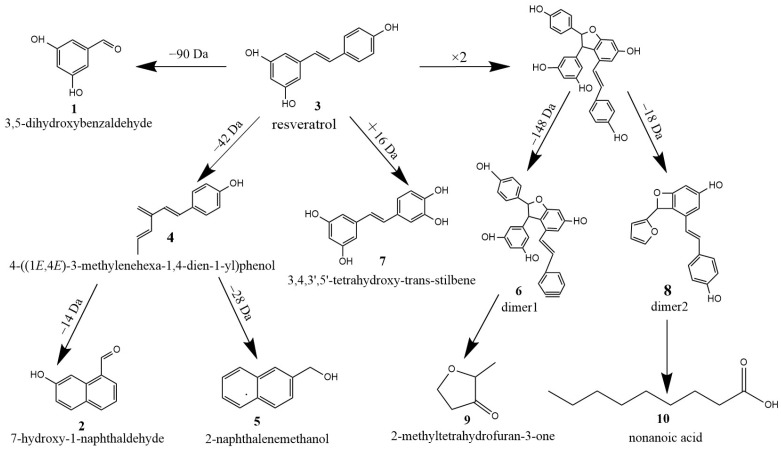
Oxidative degradation process of resveratrol.

Based on the experimental results mentioned above, a schematic diagram illustrating the degradation pathway of resveratrol is presented in Figure 8.

## 4. Conclusions

In this study, the antioxidant capacity, degradation pattern and the thermal oxidation products of resveratrol in soybean oil were evaluated. Resveratrol has demonstrated excellent in vitro antioxidant capacity. Additionally, research indicates that the addition of 600 µg/g of resveratrol has the most significant positive effect on the thermal stability of soybean oil. In this study, the HPLC-ESI-MS method and GC-MS analysis were used to identify the oxidation products produced in oil samples with exogenous addition of resveratrol during heating in an oven at 60 °C and frying at 180 °C. A total of seven non-volatile oxidation products and two volatile oxidation products were detected. The results of this study provide a theoretical basis for future research and the development of functional oils with the addition of resveratrol.

## Figures and Tables

**Table 1:** Retention time and ESI pyrolysis mode of each non-volatile oxidation product by mass spectrometry.

Peak	T (min)	Major Fragments ESI Negative/Positive		
		[M + H]^+^	[M − H]^−^	Production Scan Experiments
1 (3,5-dihydroxybenzaldehyde)	5.2		138.03	227→138
2 (7-hydroxy-1-naphthaldehyde)	6.7		171.04	227→185→171
3 (resveratrol)	7.9		227.08	
4 (4-((1E,4E)-3-methylenehexa-1,4-dien-1-yl)phenol)	9.0		185.10	227→185
5 (2-naphthalenemethanol)	12.7		157.07	227→185→157
6 (dimer1)	13.4	436.13		453→436
7 (3,4,3′,5′-tetrahydroxy-trans-stilbene)	14.3		244.07	228→244
8 (dimer2)	17.2	306.09		453→306

Dimer1,(E)-5-(4-(2-(cyclohexa-1,5-dien-3-yn-1-yl)vinyl)-6-hydroxy-2-(4-hydroxyphenyl)-2,3-dihydrobenzofuran-3-yl)benzene-1,3-diol. Dimer2,(E)-8-(furan-2-yl)-2-(4-hydroxystyryl)-7-oxabicyclo[4.2.0]octa-1(6),2,4-trien-4-ol.

## Data Availability

The data are contained within this article and the Supplementary Materials.

## Supplementary Materials

The following supporting information can be downloaded at: https://www.mdpi.com/article/10.3390/foods13010029/s1, Figure S1: Mass spectrum of each non-volatile oxidation product of resveratrol.
