# Supplementary material for: Study of the Antioxidant Capacity and Oxidation Products of Resveratrol in Soybean Oil
Source: Foods. 2023 Dec 20;13(1):29. doi: 10.3390/foods13010029 (PMC10778236; doi:10.3390/foods13010029)
Supplement: Supplementary file 1 [file foods-13-00029-s001.zip › foods-2775210-supplementary.pdf]

Supplementary Materials

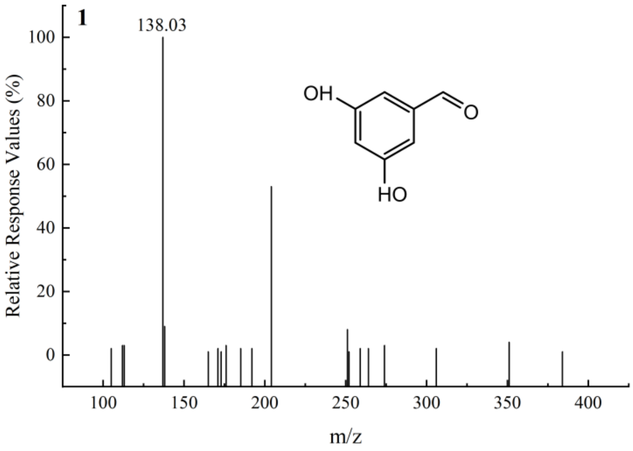

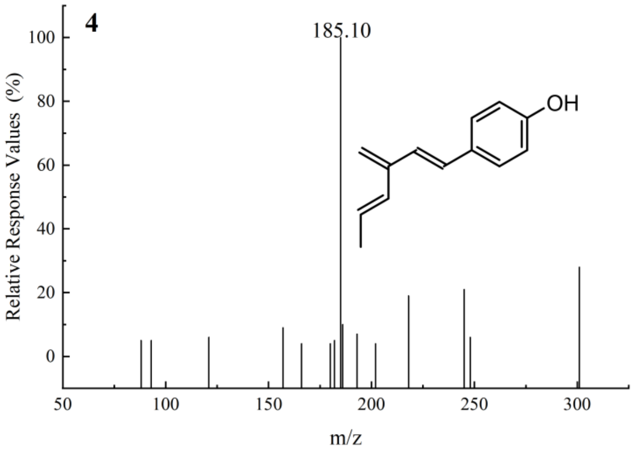

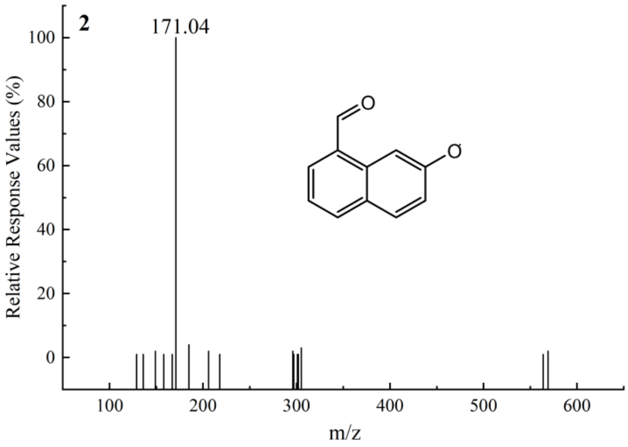

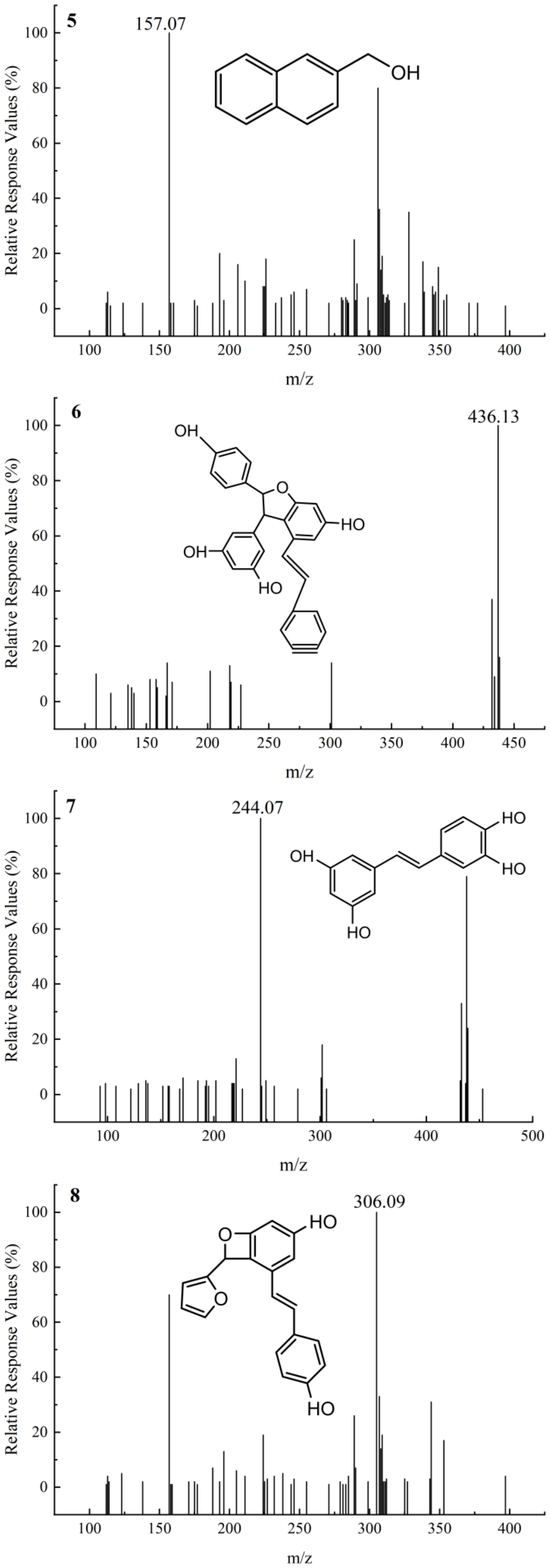

Figure S1. Mass spectrum of each non-volatile oxidation product of resveratrol.
